# A nationwide retrospective study of the seroprevalence and risk factors of transfusion‐transmissible infections among blood donors in Nigeria

**DOI:** 10.1111/tme.13160

**Published:** 2025-07-07

**Authors:** Adaeze Oreh, Felix Biyama, Tariere Bozegha, Joshua Fapohunda, Ifeanyi Mgbachi, Victoria Dalyop, Joy Elisha, Folashade Olupitan, Audu Isaiah, Catherine Babalola, Abdullahi Malammadori, Emmanuel Agahiu, Deborah Kure, Caroline Imonikhe, Chinonso Elesie, Ajoke E. Ogedegbe, Ukinebo Omokaro, Jerry Egbeaso, Chika Oparah, Omale Amedu, Maarten Postma, Theresa Nwagha, Marinus van Hulst

**Affiliations:** ^1^ Department of Planning, Research and Statistics National Blood Service Agency Abuja Nigeria; ^2^ Department of Health Sciences University Medical Centre Groningen Groningen Netherlands; ^3^ Department of Health Sciences University of Groningen Groningen Netherlands; ^4^ Data Analysis and Programme Evaluation Dataneser Consult Abuja Nigeria; ^5^ Department of Haematology Lagos State University Teaching Hospital Ikeja Nigeria; ^6^ Department of Laboratory Services Federal Medical Centre Gusau Nigeria; ^7^ Department of Pathology Federal Medical Centre Nguru Nigeria; ^8^ Blood Bank Unit Nisa Premier Hospital Abuja Nigeria; ^9^ Department of Haematology and Blood Transfusion University of Benin Teaching Hospital Benin City Nigeria; ^10^ Center of Excellence for Pharmaceutical Care Innovation Universitas Padjadjaran Bandung Indonesia; ^11^ Department of Haematology University of Nigeria Teaching Hospital Ituku‐Ozalla Nigeria; ^12^ Department of Clinical Pharmacy and Toxicology Martini Hospital Groningen Netherlands

**Keywords:** Africa, blood donors, Nigeria, seroprevalence, transfusion‐transmissible infections

## Abstract

**Background:**

Blood transfusion's life‐saving potential is often marred by the risks of transfusion‐transmissible infections (TTIs) from blood donors, for which sub‐Saharan African countries record some of the highest burdens.

**Aims:**

We aimed to assess the seroprevalence of HBV, HCV, HIV, and *Treponema pallidum* among blood donors in Nigeria, and determine the association of seropositivity with particular blood donor characteristics.

**Methods:**

A retrospective cross‐sectional study was conducted to determine the seroprevalence of HBV, HCV, HIV, and *Treponema pallidum* among blood donors in 13 blood establishments in Nigeria's six geopolitical zones from January 2018 to December 2019 following screening with highly sensitive Enzyme‐Linked Immunosorbent Assays. Data was collected from the country's web‐based software District Health Information System, Version 2 and analysed using R Studio.

**Results:**

The overall TTI seroprevalence was 10.1%, and declined from 10.9% in 2018 to 9.4% in 2019. Male donors (AOR = 0.1; 95% CI: 0.1–0.2, *p* < 0.001), those aged 46–55 years (AOR = 0.8, 95% CI: 0.7–0.9, *p* < 0.001), and first‐time donors (AOR = 0.1, 95% CI: 0.10–0.12, *p* < 0.001) were less likely to be seropositive; whereas paid donors (AOR = 2.3, 95% CI: 2.1–2.6, *p* < 0.001) and mobile blood drive donors (AOR = 1.4; 95% CI: 1.3–1.5, *p* < 0.001) were more likely to be seropositive.

**Conclusion:**

The seroprevalence of TTIs in Nigerian blood donors is high, especially among females, paid donors, and those at mobile donation sites, emphasising the importance of targeted continuous population health education and quality donor selection towards enhancing blood safety.

## 1 INTRODUCTION

Blood transfusion remains a vital therapeutic option, which saves millions of people at risk of dying from critical diseases and following life‐threatening blood loss.^1^ In Nigeria, the high frequency of road traffic accidents, obstetric blood loss, violent trauma, surgical blood loss, and anaemia from various causes drives the demand for safe blood transfusion.^2^ Despite the benefits of blood and blood products, unsafe blood transfusion remains a challenge and could result in transfusion‐transmissible infections (TTI) such as hepatitis B virus (HBV), hepatitis C virus (HCV), human immunodeficiency virus (HIV), and *Treponema pallidum*.^3^, ^4^, ^5^

The major sources of donated blood in Nigeria are paid/commercial blood donors (PDs), replacement donors (RDs), and voluntary blood donors (VDs).^1^ Patients and their families often engage PDs for a pre‐negotiated fee, whilst RDs are usually family, friends, colleagues, or acquaintances of the patient.^6^ Conversely, VDs altruistically donate blood for transfusion to unknown patients and are reportedly the safest group of blood donors due to healthier lifestyle choices and behaviours.^2^ To enhance blood safety in Nigeria, the Federal Ministry of Health and National Blood Service Agency (NBSA) have encouraged blood establishments to meet World Health Organisation (WHO) goals of 100% voluntary blood donation, criminalised commercial blood donations,^1^, ^7^, ^8^ and mandated the use of Enzyme‐linked Immunosorbent Assays as the minimum standard of screening for transfusion.^9^ However, some facilities continue to screen with rapid kits.^9^ Studies on TTI seroprevalence in Nigeria have been conducted in some states, but national data on the prevalence of TTIs among blood donors is unavailable. Therefore, our study aims to assess the seroprevalence of HBV, HCV, HIV, and *Treponema pallidum* among Nigerian blood donors and determine the association of seropositivity with blood donor characteristics. These findings will help improve strategies on donor recruitment, retention, laboratory screening, and testing strategies at blood establishments to enhance transfusion blood safety in the country.

## 2 MATERIALS AND METHODS

### 2.1 Study setting and sample size

Nigeria's blood services are regulated and coordinated by NBSA, which operates Zonal Blood Centres in the country's 6 geopolitical zones – South–South, South‐East, South‐West, North‐Central, North‐East, and North‐West, in addition to the Operational Centre in the Federal Capital Territory. Hospital‐based blood establishments also provide blood services nationwide. In these settings, blood is donated by VDs, RDs, and PDs. This study was conducted in 13 blood establishments located in the different regions.

### 2.2 Study design

A retrospective cross‐sectional study design was used to collect data on blood donors in 2018 and 2019. From the District Health Information System version 2 (DHIS2) electronic database, variables including age, sex, marital status, donor type (voluntary/replacement/commercial), donation site (fixed/mobile), number of donations (first‐time/repeat/regular), and seropositivity of HBV, HCV, HIV and/or *Treponema pallidum* were collected and cross‐checked with the BE blood donation registers.

### 2.3 Study population

Records of all blood donors registered and screened in the 13 blood establishments over the study period were included in the study. According to NBSA blood donor pre‐donation screening criteria, those qualified to donate blood are aged 18–65 years, weigh ≥ 50 kg, have a haemoglobin concentration of ≥ 12.5 g/dL, pulse rate 50–100 beats/minute, systolic blood pressure 90–140 mmHg, diastolic blood pressure 60–100 mmHg, and no known medical illness.^9^ Inclusion criteria for hospital blood banks included status as a referral hospital, a minimum of 100 patient bed spaces, provision of secondary and/or tertiary health services, and facilities for laboratory screening of blood for HIV, HBV, HCV, and syphilis with ELISA. Exclusion criteria, which included poor routine data reporting and TTI laboratory screening with rapid test kits, led to the exclusion of 21 establishments.

### 2.4 Laboratory screening methods

First, screening for HIV, HBV, HCV, and syphilis was done using highly sensitive ELISA (Appendix A). All positive samples were subjected to a second ELISA test. If the outcome was reactive following both tests, then the result was marked seropositive. Discordant samples were recorded as indeterminate. Standard operating procedures (SOPs) for blood screening and result interpretation were adhered to in all included laboratories according to the equipment manufacturer instructions.

### 2.5 Data management and analysis

The DHIS2, a real‐time web‐based software on Nigeria's National Health Management Information System, has been operational since 2010. In 2018, with support from WHO Nigeria Country Office, NBSA adopted and customised DHIS2 as its primary digital platform for collecting blood services data from BEs across the country. All data generated through DHIS2 is owned by the Federal Ministry of Health, processed, and stored in a central server at the headquarters in Abuja, Nigeria, and blood donor data per facility are routinely sent to the platform monthly. The data are entered by trained haematologists, medical laboratory scientists, or scientific officers who have been extensively trained on data entry and use of DHIS2. Data managers at the NBSA headquarters subsequently clean the data regularly for duplicates and missing variables. In this study, only data collected on the most recent visit of repeat donors were collected to avoid double counting. A checklist capturing the relevant variables was developed to extract data from DHIS2, and the data was analysed using RStudio v2022.07.2 + 576.pro12.^10^

Donor characteristics were described as percentages, and associations of prevalence of HBV, HCV, HIV, *Treponema pallidum* and the explanatory variables were tested. Study variables found to be statistically significant (*p* ≤ 0.05) on bivariate analysis using the Pearson's Chi‐square test were then subjected to logistic regression. For each of the TTIs and explanatory variables, the findings from logistic regression were expressed as estimated (crude) odds ratios (ORs) and adjusted odds ratios (AORs) after adjusting for the effect of potential confounders with 95% confidence intervals (CIs).

### 2.6 Ethical clearance

Permission was obtained from NBSA to access raw blood donation data from DHIS2. As this research evaluated secondary data, additional donor consent distinct from that obtained pre‐donation was not required. However, ethical clearance was obtained from the National Human Research and Ethics Committee (NHREC) of the Federal Ministry of Health (NHREC/01/01/2007‐06/09/2023). All data was fully anonymised to assure donor data privacy and confidentiality.

## 3 RESULTS

### 3.1 Socio‐demographic characteristics of the blood donors

A total of 98 610 donations at 13 blood establishments between January 2018 and December 2019 were screened for TTIs using ELISA. Only 2.8% of the blood donors were female, and nearly 70% of all blood donors were aged 18–35 years (Table 1).

**TABLE 1:** Seroprevalence of transfusion‐– transmissible infections by age group, sex, marital status, donor type, number of donations, and donation site.

Variable	Category	Number of donors (*N* = 98 610)	Number of Seropositive Donors	Percentage of all donors (%) [95% CI]	Percentage of donors seropositive (%) [95% CI]	Percentage of donors seropositive for HBV (%) [95% CI]	Percentage of donors seropositive for HCV (%) [95% CI]	Percentage of donors seropositive for HIV (%) [95% CI]	Percentage of donors seropositive for *Treponema pallidum* (%) [95% CI]
Overall (2018–2019)	All donors	98 610	10 015	100	10.1 [9.9–10.3]	5.6 [5.5–5.7]	2.4 [2.3–2.4]	1.2 [1.1–1.2]	0.9 [0.8–0.9]
2018	All donors	43 148	4752	100	10.9 [10.7–11.3]	6.0 [5.7–6.1]	2.6 [2.4–2.7]	1.3 [1.2–1.4]	1.1 [1.0–1.2]
2019	All donors	55 462	5263	100	9.4 [9.2–9.7]	5.4 [5.2–5.5]	2.2 [2.1–2.3]	1.1 [0.9–1.1]	0.8 [0.7–0.8]
Age group	18–25 years	32 391	2796	32.9 [32.5–33.1]	8.6 [8.3–8.9]	5.2 [4.9–5.4]	2.0 [1.7–2.3]	0.9 [0.8–0.9]	0.5 [0.4–0.6]
	26–35 years	36 147	3835	36.7 [36.3–36.9]	10.6 [10.2–10.9]	5.6 [5.3–5.9]	2.5 [2.2–2.8]	1.3 [1.2–1.4]	1.1 [1.0–1.2]
	36–45 years	16 304	2071	16.5 [16.3–16.8]	12.7 [12.1–13.2]	6.9 [6.5–7.3]	3.1 [2.5–3.5]	1.6 [1.4–1.8]	1.0 [0.9–1.2]
	46–55 years	10 690	843	10.8 [10.6–11.0]	7.9 [7.3–8.3]	4.3 [3.9–4.7]	1.8 [1.2–2.2]	0.9 [0.7–1.1]	0.8 [0.6–1.0]
	56–65 years	3078	470	3.1 [3.0–3.2]	15.3 [13.9–16.5]	8.3 [7.3–9.2]	3.7 [2.3–4.9]	1.5 [1.0–1.9]	1.7 [1.2–2.2]
Sex	Female	2750	1250	2.8 [2.6–2.8]	45.5 [43.5–47.3]	22.9 [21.4–24.5]	11.6 [10.4–12.7]	5.9 [5.0–6.8]	4.9 [4.1–5.7]
	Male	95 860	8765	97.2 [97.1–97.3]	9.1 [8.9–9.3]	5.1 [5.0–5.2]	2.1 [2.0–2.2]	1.0 [0.9–1.1]	0.8 [0.0–0.8]
Marital status	Single	39 023	4041	39.6 [39.2–39.8]	10.3 [10.0–10.6]	5.7 [5.5–5.9]	2.5 [2.3–2.6]	1.1 [1.0–1.2]	0.9 [0.8–1.0]
	Married	49 004	4894	49.7 [49.3–50.0]	9.9 [9.7–10.2]	5.5 [5.3–5.7]	2.3 [2.2–2.4]	1.2 [1.0–1.2]	0.9 [1.0–1.2]
	Divorced	8682	871	8.8 [8.6–8.9]	10.0 [9.4–10.6]	5.4 [4.9–5.8]	2.2 [1.9–2.5]	1.1 [1.0–1.4]	0.9 [0.8–1.4]
	Widowed	1901	209	1.9 [1.8–2.0]	10.9 [9.5–12.4]	6.9 [5.8–8.1]	2.2 [1.5–2.8]	1.2 [0.7–1.7]	0.5 [0.7–1.7]
Donor type	Replacement	54 400	3610	55.2 [54.8–55.4]	6.6 [6.4–6.8]	2.9 [2.8–3.0]	1.5 [1.4–1.6]	1.2 [1.1–1.3]	0.9 [0.8–0.9]
	Voluntary	44 097	6375	44.7 [44.4–45.0]	14.5 [14.1–14.7]	8.9 [8.7–9.2]	3.4 [3.2–3.5]	1.1 [1.0–1.2]	0.8 [0.8–1.0]
	Paid	113	30	0.1 [0.09–0.1]	26.5 [18.2–34.8]	9.7 [4.1–15.2]	4.4 [0.5–8.2]	11.5 [5.5–17.4]	0.8 [0.8–2.6]
Number of donations	One	95 188	8436	96.5 [96.4–96.6]	8.8 [8.6–9.0]	4.9 [4.8–5.1]	2.1 [2.0–2.1]	1.0 [0.9–1.0]	0.7 [0.6–0.7]
	Two	1022	448	1.1 [0.9–1.1]	43.8 [40.7–46.8]	23.9 [21.3–26.5]	9.2 [7.5–11.0]	5.7 [4.3–7.2]	4.7 [3.4–6.1]
	More than two	2400	1131	2.4 [2.3–2.5]	47.1 [45.1–49.1]	24.0 [22.3–25.7]	11.1 [9.8–12.3]	5.3 [4.4–6.2]	6.6 [5.6–7.6]
Donation site	Fixed	58 783	5038	59.6 [59.3–59.9]	8.5 [8.3–8.7]	4.1 [3.9–4.2]	2.0 [1.8–2.1]	1.3 [1.2–1.4]	1.0 [0.9–1.1]
	Mobile	39 827	4977	40.4 [40.0–40.6]	12.4 [12.1–12.8]	7.8 [7.6–8.1]	2.9 [2.8–3.1]	0.9 [0.8–0.9]	0.7 [0.6–0.8]

Abbreviations: CI, confidence interval; HBV, hepatitis B virus; HCV, hepatitis C virus; HIV, human immunodeficiency virus.

Most donors (96.5%) were first‐time donors, and only 3.5% could be referred to as repeat (1.1%) or regular donors (2.4%) (having donated more than twice). The majority were replacement donors (RD) (55.2%); 44.7% were voluntary blood donors, and 0.1% were self‐declared paid donors. Most donations occurred at fixed blood donation sites (59.6%), while 40.4% occurred at mobile blood drives.

### 3.2 Seroprevalence of TTIs among blood donors

Among 98 610 donors studied, 10.1% were seropositive for at least one TTI (Table 1). Seropositivity rates for HBV, HCV, HIV, and *Treponema pallidum* were 5.6%(CI: 5.5–5,7), 2.4%(CI: 2.3–2.4), 1.2%(CI: 1.1–1.2), and 0.9%(CI: 0.8–0.9) respectively.

HBV was the most prevalent TTI in both 2018 and 2019. Multiple infections were seen in 0.2% of donors (Maximum = 2; HBV/HCV and HBV/Syphilis). The TTI frequency was comparatively higher in 2018 than in 2019 (10.9% vs. 9.4%, *p* < 0.001), and in female donors compared to males (45.5% vs. 9.1%, *p* < 2.2e‐16). Additionally, for each TTI, female donors had higher frequencies than males— HBV (22.9% vs. 5.1%), HCV (11.6% vs. 2.1%), HIV (5.9% vs. 1.0%), and syphilis (4.9% vs. 0.8%). Mixed infections (MI) of HBV with HCV or syphilis were only observed in two married male donors aged 18–25 years and 26–35 years respectively.

The frequency of TTIs was highest in donors aged 56–65 years (15.3%) and 36–45 years (12.7%). HIV was more prevalent in donors aged 36–45 years (1.6%), while HBV, HCV, and *Treponema pallidum* were most seroprevalent in donors aged 56–65 years (8.3%, 3.7% and 1.7% respectively). Donors aged 18–25 years and 46–55 years had the least seropositivity rates. The highest frequencies of TTIs were observed in paid donors (26.5%) compared to 14.5% in voluntary donors, 6.6% in replacement donors, those who had donated more than twice (47.1%), and donors from mobile drives (12.4%).

**FIGURE 1:**
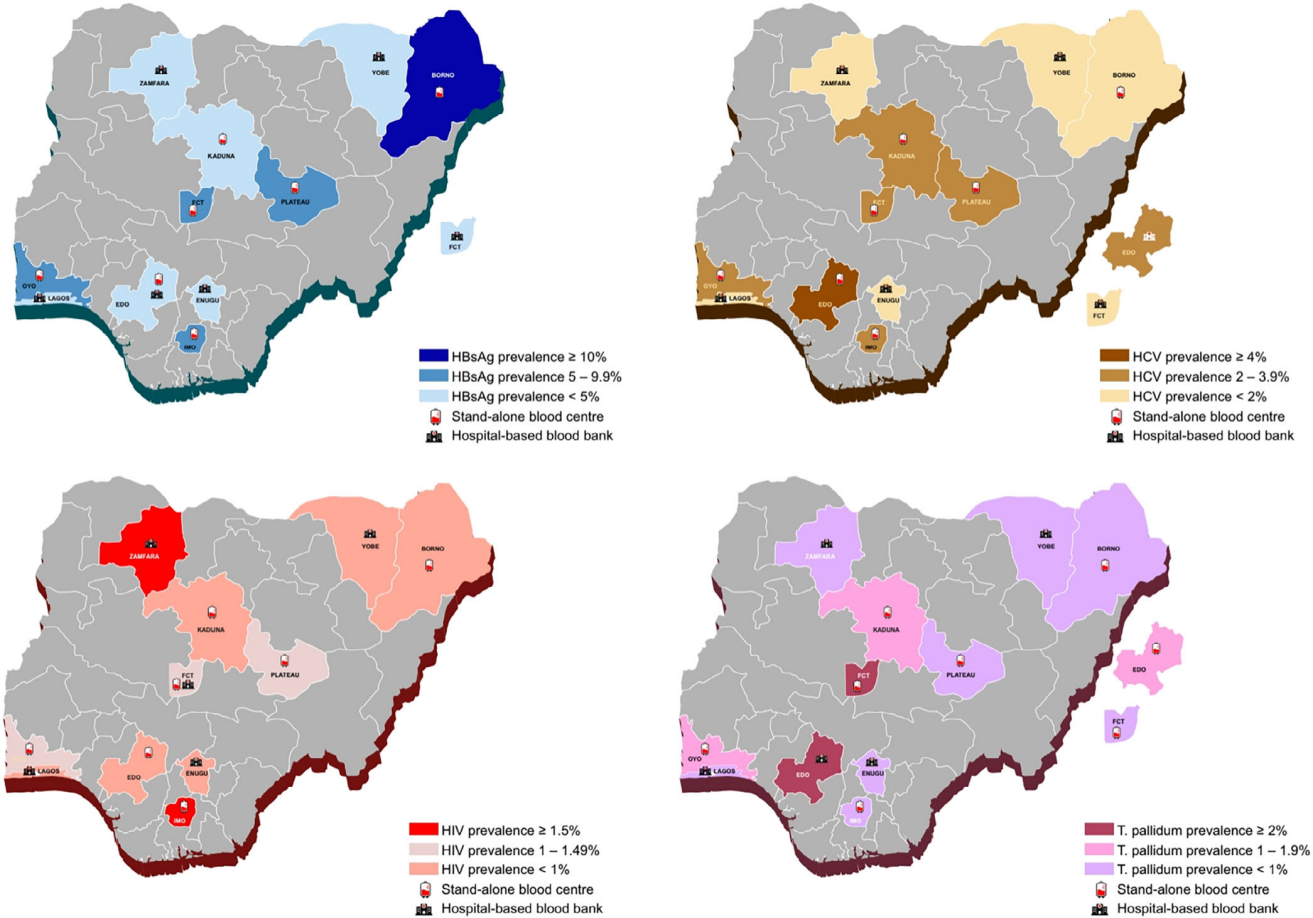
Spatial distribution of transfusion transmissible infections Hepatitis B, Hepatitis C, Human Immunodeficiency Virus (HIV) and *Treponema pallidum* in Nigeria's six geopolitical regions.

On bivariate analysis, the relationships between age, sex, donor type, number of blood donations, donation site, and TTI seropositivity were all statistically significant [X^2^(*df* = 4, *N* = 98 610) = 355.03, *p*‐value <2.2e‐16; X^2^(*df* = 1, N = 98 610) = 3858.9, *p*‐value <2.2e‐16; X^2^(*df* = 2, N = 98 610) = 1665.8, *p*‐value <2.2e‐16; X^2^(*df* = 2, N = 98 610) = 5039.8, *p*‐value <2.2e‐16; X^2^(*df* = 1, N = 98 610) = 401.05, *p*‐value <2.2e‐16 respectively]. Regional variations were also observed in the seropositivity of specific TTIs (Figure 1).

### 3.3 Association between donor characteristics and TTI seropositivity

Multivariate logistic regression analysis revealed the associations between donor characteristics and TTI seropositivity (Table 2). Male donors (AOR = 0.1; 95% CI: 0.1–0.2, *p* < 0.001) were less likely than female donors to be TTI seropositive. Additionally, compared to 18–25‐year‐old donors, other age groups were more likely to be seropositive except 46–55‐year‐olds who were less likely to be TTI seropositive (AOR = 0.8, 95% CI: 0.7–0.9, *p* < 0.001). Furthermore, mobile blood drive donors (AOR = 1.4; 95% CI: 1.3–1.5, *p* < 0.001) were more likely than fixed site donors to be seropositive. Also, PDs were two times more likely to be seropositive than VDs (AOR = 2.3, 95% CI: 2.1–2.6, *p* = 0.002). Compared to donors who had donated blood more than twice, first‐time donors were less likely to be seropositive (AOR = 0.1, 95% CI: 0.10–0.12, p < 0.001) (Table 2).

**TABLE 2:** Multivariate analysis of the association between TTI seropositivity and donor characteristics.

		Positive for TTIs			
Donor characteristic		Crude odds ratio (COR) (95% CI)	*P*–value	Adjusted odds ratio (AOR) AOR (95% CI)	*P*–value
Age group	18–25 years	1.0		1.0	
	25–35 years	1.2 (1.1–1.3)	< 0.001	1.2 (1.1–1.3)	< 0.001
	36–45 years	1.5 (1.4–1.6)	< 0.001	1.4 (1.3–1.5)	< 0.001
	46–55 years	0.9 (0.8–0.9)	0.01	0.8 (0.7–0.9)	< 0.001
	56–65 years	1.9 (1.7–2.1)	< 0.001	1.7 (1.5–1.9)	< 0.001
Sex	Female	1.0		1.0	
	Male	0.1 (0.1–0.1)	< 0.001	0.1 (0.1–0.2)	< 0.001
Marital status	Divorced	1.0		1.0	
	Married	0.9 (0.9–1.0)	0.89	0.9 (0.9–1.0)	0.94
	Single	1.0 (0.9–1.1)	0.37	1.0 (0.9–1.1)	0.09
	Widowed	1.1 (0.9–1.2)	0.2	1.2 (1.0–1.4)	0.02
Donation site	Fixed site	1.0		1.0	
	Mobile site	1.5 (1.4–1.6)	< 0.001	1.4 (1.3–1.5)	< 0.001
Donor type	VNRD	1.0		1.0	
	RD	0.4 (0.4–0.4)	< 0.001	0.01 (0.009–0.01)	< 0.001
	PD	2.1 (1.4–3.2)	< 0.001	2.3 (2.1–2.6)	0.002
Number of donations	Greater than 2	1.0		1.0	
	One	0.1 (0.1–0.1)	< 0.001	0.1 (0.10–0.12)	<0.001
	Two	0.8 (0.7–1.0)	0.07	0.9 (0.7–1.1)	0.26
Regional location	NW hospital centre	1.0		1.0	
	NC blood centre I	2.1 (1.9–2.4)	< 0.001	0.6 (0.5–0.7)	< 0.001
	NC blood centre II	2.3 (2.1–2.5)	< 0.001	0.1 (0.07–0.14)	< 0.001
	NE blood centre	1.8 (1.5–2.2)	< 0.001	0.05 (0.03–0.08)	< 0.001
	NW blood centre	0.9 (0.7–1.12)	< 0.001	0.02 (0.01–0.03)	< 0.001
	SE blood centre	1.3 (1.0–1.8)	0.02	0.06 (0.03–0.09)	< 0.001
	SS blood centre	1.0 (0.8–1.2)	0.9	0.02 (0.01–0.03)	< 0.001
	SW blood centre	1.7 (1.5–1.9)	0.4	0.04 (0.03–0.06)	< 0.001
	NC hospital centre	0.3 (0.2–0.4)	< 0.001	0.1 (0.09–0.2)	< 0.001
	NE hospital centre	0.7 (0.6–0.8)	< 0.001	0.5 (0.4–0.5)	< 0.001
	SE hospital centre	0.6 (0.5–0.7)	< 0.001	0.6 (0.5–0.7)	< 0.001
	SS hospital centre	1.7 (1.6–1.9)	< 0.001	1.5 (1.4–1.7)	< 0.001
	SW hospital centre	0.4 (0.3–0.5)	< 0.001	0.4 (0.4–0.5)	< 0.001

Abbreviations: CI, confidence interval; NC, North–Central; NE, North–East; NW, North–West; PD, paid donors; RD, replacement donors; SE, South–East; SS, South–South; SW, South–West; TTI, transfusion‐transmissible infection; VNRD, voluntary non‐remunerated donors.

## 4 DISCUSSION

We assessed HBV, HCV, HIV, and syphilis seroprevalence among blood donors in 13 blood establishments across Nigeria's six geopolitical regions between 2018 and 2019. The overall prevalence was 10.1% (*N* = 10 015) with regional differences observed in prevalence rates. These findings were below prior Nigerian prevalence figures ranging from 11.9% to 14.9%.^7^, ^8^ We also observed a decline in prevalence from 2018(10.9%) to 2019(9.4%) [*p* < 0.001]. Enhanced awareness of TTI risks, blood donor eligibility criteria, and personal donor risk assessment could lead to self‐deferral accounting for these declines.^11^, ^12^

Sex was a significant determinant of TTI seropositivity, with females more likely to have TTI markers. Despite only 2.8% of study donors being female, they had markedly high TTI seropositivity rates, in contrast to past Eritrean and Ethiopian research.^11^, ^12^ In Tanzania, however, perhaps due to similarities in access to health education and screening programmes, no significant difference in TTI seropositivity was observed between sexes.^13^ In Africa, females make up a very low proportion of blood donors. There is therefore a need for higher blood donor recruitment and retention in Nigerian female populations to expand the blood donor pools. However, enhancing their participation would only be beneficial if their donations are considered safe. It is therefore paramount that to create safer blood donor pools and given the vulnerability of women to TTIs, gender‐specific strategies such as targeted health educational interventions, perhaps leveraging technology to enhance self‐care, provoke lifestyle changes, and enhance treatment adherence be implemented.^14^, ^15^ The higher likelihood of women to be seropositive for TTIs is not restricted to donor populations. Available data reveals that in the general population, women are more likely than their male counterparts to be living with infections such as HIV.^16^ Biological, religious, cultural, and socio‐economic factors all contribute distinctly to the increased vulnerability of women to TTIs.^17^, ^18^ In fact, the 2018 Nigeria HIV/AIDS Indicator and Impact Survey (NAIIS), a national household‐based survey that assessed the prevalence of HIV and related health indicators, revealed nearly double HIV prevalence rates in women across all age groups surveyed.^19^

Blood donors aged 46–55 years had lower seropositivity rates compared to those aged 18–25 years, which may reflect behavioural differences and effects of immunisation policies on specific age groups. We also observed TTI seropositivity to be more likely in mobile drive donors compared to those from fixed donation locations, thus demonstrating the importance of donation site selection for quality blood services.^20^

Replacement donors were less likely than VDs to be TTI seropositive. It is likely that patients and their families engage replacement blood donors based on the perceived safe lifestyle behaviours of their relatives and acquaintances. To improve transfusion safety, the conversion of RDs to VDs through centralised blood transfusion services has been advocated.^21^ This has however been controversial due to the constraints this strategy may impose on already existing unmet blood needs in SSA.^22^ Malawi, for example, reported sharp declines in per capita blood donations between 2011 and 2014 following the adoption of this policy, resulting in two‐thirds of the national transfusion need being unmet.^23^ In our study, only 1.1% and 2.4% of donors were repeat and regular donors respectively, illustrating the low rates of donor retention in Africa.^24^, ^25^

First‐time donors were less likely to be TTI seropositive than regular donors who had donated blood more than twice. This contrasts the ‘high‐risk’ perception of first‐time donors, and highlights opportunities to enhance Nigeria's blood donor base through consistent community health education, and effective blood donor retention strategies, irrespective of donor type or number of prior donations. The higher rates in repeat and regular donors, raise important questions about the use of paid donors as a source of blood for transfusion, which by virtue of the National Blood Service Commission Act of 2021 is punishable by law.^9^ Additionally, it calls into question the quality of pre‐donation counselling that is given to repeat and regular donors, who may frequently put themselves at risk if healthy behaviours are not reiterated on their visits.

The overall HBV seroprevalence in this study was 5.6% (95% CI: 5.5–5.8). The North‐East regional blood centre recorded the highest seropositivity for HBV markers in this study (11.0%), while the least frequency was observed in the North‐Central hospital centre (0.9%). Nigeria belongs to the HBV hyperendemic zone, and previous studies have revealed seropositivity rates ranging between 1.0% and 28.4% among various population sub‐groups.^26^, ^27^, ^28^ In 2014, general population prevalence of HBV in South‐West Nigeria was as high as 16.3%, but averaged 4.6% in the same region in our study. The increasing uptake of HBV vaccination, which was introduced into Nigeria's routine vaccination schedule in 2004, may be contributory to lower prevalence rates among blood donors in certain regions.^29^, ^30^, ^31^ Seroprevalence variations may also reflect socio‐cultural lifestyle and behaviour differences, population risks, and endemicity.^12^, ^32^

In our study, HCV seropositivity was 2.3% (95% CI: 2.3–2.4), compared to a previous general population seropositivity rate of 1.3%.^33^ Other previous findings of HCV prevalence were however higher in South–South Nigeria and North‐Central Nigeria, ranging between 3.6% and 4.1% respectively.^7^, ^8^ However, our findings exceed those reported earlier from Port Harcourt (0.5%), Enugu (0.9%), and Nnewi (2.0%).^33^, ^34^, ^35^ This points at possibly increasing prevalence across different regions in Nigeria. It is also worth mentioning that in African populations, HCV antibody tests are associated with a high frequency of false positive antibodies due to malaria, *Schistosoma mansoni*, syphilis, HIV, malnutrition, or other chronic infections.^36^, ^37^ Thus, in the absence of confirmatory nucleic acid‐based testing (NAT) for HCV, findings based on ELISA testing cannot be reported with any level of certainty.^11^

A HIV seropositivity of 1.2% (95% CI: 1.1–1.3) was recorded among blood donors, lower than reports from Burkina Faso, Equatorial Guinea, and Tanzania, but exceeding findings from Eritrea and Ethiopia.^11^, ^12^, ^13^, ^32^, ^38^ This, however, closely approximates the national population prevalence of 1.4% reported by the Nigerian NAIIS household survey 2018 and corresponds with findings in South African donors. However, HIV prevalence in South Africa's donors is 18 times lower than the general population.^19^, ^39^ There is thus much room for improvement in blood donor selection and transfusion safety in Nigeria.

An overall *Treponema pallidum* seropositivity of 0.9% (95% CI: 0.8–1.0) was reported in our study. Female donors (4.9%; 95% CI: 4.2–5.8) were more frequently seropositive compared to male donors (0.9%; 95% CI: 0.7–0.9). Whereas this prevalence is substantially lower than previous findings from Burkina Faso (2.4%),^39^ it closely corresponds to Eritrean, Ethiopian, and Kenyan findings.^11^, ^12^, ^40^ The highest prevalence was observed in the North‐Central blood centre (3.0%) and South–South region hospital centre (2.5%) in keeping with some past Nigerian studies.^1^, ^7^ Additionally, donors aged 56–65 years had almost double the seropositivity for *Treponema pallidum* compared to other age groups (Table 1), corroborating previous reports of higher prevalence in older donors.^41^

### 4.1 Study limitations

As a retrospective study, information on several factors, including occupational data, which may have influenced our findings, could not be obtained. Additionally, customised screening questionnaires may have highlighted opportunities to optimise contextually relevant donor selection and testing algorithms.

The lack of confirmatory testing and the intrinsic weakness of ELISA techniques in contrast to NAT were other limitations.^42^, ^43^ Therefore, our study may under‐report (due to the presence of window period) or overestimate (high rate of HCV false positivity) TTI frequency among donors. Furthermore, as first‐time donors constituted the majority, repeat donors who would ideally provide a proxy for incidence,^44^ could not be used to determine TTI incidence rates and thus ascertain residual risk.^45^, ^46^, ^47^ Also, especially in hospital‐based settings, seropositive donors may not be linked to specialist care services, and studies from Ghana and Tanzania have reported low rates of notification and linkage to care from transfusion services.^13^, ^48^, ^49^ Post‐donation TTI counselling and follow‐up care are vital for preventing future donation by seropositive donors, enabling early clinical treatment of the infection, minimising disease impact and the risk to their partners and close contacts, reducing new cases of TTIs, thus protecting multiple lives. It is therefore beneficial not simply to the blood donation centre and the blood donor but to the community.^50^

Despite our study finding a high TTI seroprevalence, this cannot translate to infection risk, as some data required for successful infection risk modelling were unavailable. Despite the availability of infection prevalence data and percentage of first‐time and repeat donors, the low proportion of regular donors (2.4%), negligible data on inter‐donation intervals, and unavailable data on the average age of transfusion recipients did not permit the determination of TTI incidence and risk.^51^

Significant regional differences were identified in this study. This likely demonstrates a need for more effective donor selection, testing, and retention strategies in Nigeria and across the continent. However, further studies could determine whether these observations were due to geographical, socio‐cultural, or behavioural differences, or purely variations in donor selection, testing, or retention strategies. Thus, providing insights which could inform targeted interventions and increase the numbers of voluntary donors. Future research could also explore the role of paid blood donors and the impact of mobile blood drives on donor recruitment, retention, and safety, particularly in rural settings.

## 5 CONCLUSION

The TTI seroprevalence in Nigerian blood donors, especially female donors, is high, with similarities between HIV prevalence in blood donors and the general population. This indicates a need for significant improvement in the selection and retention of safe donors for lower infection prevalence. We also noted other significant associations between TTI seroprevalence and age, donor type, number of donations, and donation sites.

This study contributes substantially to current knowledge of regional characteristics of blood donor seropositivity in Nigeria, which could strengthen evidence‐based policy for quality donor selection and infectious disease management.^52^ Building on our findings, health education should rely on population demographics for effective donor recruitment and retention to enhance the numbers of safe repeat and regular donors. The high TTI seroprevalence rate observed among women is particularly worrisome. As women constitute a substantial proportion of Nigeria's general population (49.5%),^53^ blood donor safety would require increased efforts aimed at reducing their exposure to TTIs, thereby enabling their safe contribution as blood donors. Intersectoral collaborations between NBSA, State and Federal Ministries of Health, National Orientation Agency and Ministry of Information could collaboratively enhance population health promotion and positive behavioural change for blood donor safety, thus reducing TTI prevalence and perhaps risk. Additionally, regardless of a blood donor's status as a repeat or regular donor, counselling at each engagement in blood establishments must be an on‐going activity, and its importance in enhancing blood safety should not be disregarded. Furthermore, although antibody assays such as ELISA are deemed cost effective and cost saving, NAT testing in Nigeria could be explored in future to enhance TTI detection capabilities and the incremental cost‐utility which has been ascribed to it.^54^

## AUTHOR CONTRIBUTIONS

Oreh A, Biyama F, Bozegha T, Fapohunda J, Dalyop V, Elisha J, Olupitan F, Isaiah A, Babalola C, Malammadori A, Agahiu E, Kure D, Imonikhe C, Elesie C, Ogedegbe AE, Omokaro U, Egbeaso J, and Oparah C Amedu O, van Hulst M, Nwagha T, and Postma M, conceived and designed the study. Oreh A, Biyama F, Bozegha T, contributed to the acquisition of these data. Oreh A, Biyama F, Bozegha T, Mgbachi I, and van Hulst M analysed the data. Oreh A, Biyama F, Bozegha T, van Hulst M, Nwagha T, and Postma M contributed to the interpretation of these data. Oreh A drafted the initial manuscript, and all authors contributed to re‐drafting and critically revising the manuscript for intellectual content. All authors provided final approval for the version to be published.

## FUNDING INFORMATION

No additional funding was provided for this research.

## CONFLICT OF INTEREST STATEMENT

The authors have no competing interests.

## PATIENT CONSENT STATEMENT

Pre‐donation and consent for research participation were obtained from all blood donors in this study.

## Data Availability

The data that support the findings of this study are available from Nigeria's National Blood Service Agency. Restrictions apply to the availability of these data due to privacy or ethical restrictions. Data are available upon reasonable request with the permission of the National Blood Service Agency.

## APPENDIX A

**Table:**

Infection	Blood establishment	Assay type
HBV	Stand‐alone blood establishments; Hospital‐based blood establishments (South‐East, South–South)	Bio‐Rad Monolisa HBsAg ULTRA Assay Kit/96 (Bio‐Rad Laboratories, USA)
	Hospital‐based blood bank establishments (South‐West)	Dialab HbSAg ELISA (Dialab GmbH, Austria)
	Hospital‐based blood establishments (North‐Central, North‐East; North‐West)	RecombiLISA HbSAg ELISA (CTK Biotech, USA)
HCV	Stand‐alone blood establishments	DIA‐PRO HCV Ab ELISA Kit/96 (Diagnostic Bioprobes Srl, Italy).
	Hospital‐based blood establishments (North‐Central, North‐East; North‐West)	RecombiLISA HCV Ab ELISA (CTK Biotech, USA)
	Hospital‐based blood establishments (South‐East, South–South)	Ortho HCV 3.0 SAVe ELISA Test System (Bio‐Rad Laboratories, USA)
	Hospital‐based blood establishments (South‐West)	Dialab HCV Ab ELISA (Dialab GmbH, Austria)
HIV 1 & 2	Stand‐alone blood establishments	Gensreen ULTRA HIV Ag/Ab ELISA Kit/96
	Hospital‐based blood establishments (South‐East, South–South)	Vironostika HIV Uni‐Form II Ag/Ab (BioMerieux, France)
	Hospital‐based blood establishments (North‐East; North‐West, North‐Central)	RecombiLISA HIV 1 + 2 Ab ELISA (CTK Biotech, USA)
	Hospital‐based blood establishments (South‐West)	Dialab HIV 1&2 Ag/Ab ELISA (Dialab GmbH, Austria)
*Treponema pallidum*	Stand‐alone blood establishments	DIA‐PRO Diagnostic Bioprobe Syphilis Ab One Version ULTRA ELISA Kit/96
	Hospital‐based blood establishments (North‐East; North‐West, North‐Central)	RecombiLISA Syphilis Ab ELISA CE (CTK Biotech, USA)
	Hospital‐based blood establishments (South‐East, South–South)	Enzyme immunoassay Eti‐syphilis‐G and Eti‐syphilis‐M (DiaSorin Diagnostics, Italy)
	Hospital‐based blood establishments (South‐West)	Dialab Syphilis IgG/IgM ELISA (Dialab GmbH, Austria)

Abbreviations: Ab, antibody; Ag, antigen; ELISA, enzyme‐linked immunosorbent assay; HbSAg, hepatitis B surface antigen; HCV, hepatitis C virus; HIV, human immunodeficiency virus; IgG, immunoglobulin G; IgM, immunoglobulin M.
